# Cannabis suppresses antitumor immunity by inhibiting JAK/STAT signaling in T cells through CNR2

**DOI:** 10.1038/s41392-022-00918-y

**Published:** 2022-04-06

**Authors:** Xinxin Xiong, Siyu Chen, Jianfei Shen, Hua You, Han Yang, Chao Yan, Ziqian Fang, Jianeng Zhang, Xiuyu Cai, Xingjun Dong, Tiebang Kang, Wende Li, Penghui Zhou

**Affiliations:** 1 State Key Laboratory of Oncology in Southern China, Collaborative Innovation Center for Cancer Medicine, Sun Yat-sen University Cancer Center, Guangzhou, 510060 China; 2 Guangdong Provincial People’s Hospital, Guangdong Academy of Medical Sciences, Guangzhou, 510515 China; 3 Guangdong Laboratory Animals Monitoring Institute, Guangdong Key Laboratory of Laboratory Animals, Guangzhou, 510663 China; 4 Department of Thoracic Surgery, Taizhou Hospital of Zhejiang Province, Wenzhou Medical University, Linhai, 317000 China; 5 Affiliated Cancer Hospital &Institute of Guangzhou Medical University, Guangzhou, 510095 China; 6 Department of Thoracic Surgery, Sun Yat-sen University Cancer Center, Guangzhou, 510060 China; 7 State Key Laboratory of Pharmaceutical Biotechnology, Nanjing University, Nanjing, 210023 China

**Keywords:** Tumour immunology, Immunology

## Abstract

The combination of immune checkpoint blockade (ICB) with chemotherapy significantly improves clinical benefit of cancer treatment. Since chemotherapy is often associated with adverse events, concomitant treatment with drugs managing side effects of chemotherapy is frequently used in the combination therapy. However, whether these ancillary drugs could impede immunotherapy remains unknown. Here, we showed that ^∆^9-tetrahydrocannabinol (THC), the key ingredient of drugs approved for the treatment of chemotherapy-caused nausea, reduced the therapeutic effect of PD-1 blockade. The endogenous cannabinoid anandamide (AEA) also impeded antitumor immunity, indicating an immunosuppressive role of the endogenous cannabinoid system (ECS). Consistently, high levels of AEA in the sera were associated with poor overall survival in cancer patients. We further found that cannabinoids impaired the function of tumor-specific T cells through CNR2. Using a knock-in mouse model expressing a FLAG-tagged *Cnr2* gene, we discovered that CNR2 binds to JAK1 and inhibits the downstream STAT signaling in T cells. Taken together, our results unveiled a novel mechanism of the ECS-mediated suppression on T-cell immunity against cancer, and suggest that cannabis and cannabinoid drugs should be avoided during immunotherapy.

## Introduction

Recent progress in immunotherapy has revolutionized cancer treatment by inducing durable responses.^1^ ICB therapies, such as CTLA4, PD-1, and PD-L1 antibodies, have been approved for the treatment of more than 50 cancer types.^2,3^ Despite that, low response rates and immune-relevant side effects have been observed.^4^ A number of drugs are frequently used during ICB therapies to manage ICB-related side effects.^5^ To improve the responses to ICB, more than 4000 clinical trials are currently testing the combination potency of ICB with other approved therapies or drugs.^6^ Although enhanced therapeutic efficacy was achieved in some trials, the co-occurrence of adverse events and toxicity was also observed, which led to the use of drugs to treat both the immune-relevant and combined drug-mediated side effects.^7,8^ However, whether these ancillary drugs interfere with the treatment of ICB remains unexplored. Avoiding or replacing drugs attenuating antitumor immunity during the ICB treatment could instantly improve clinical outcomes of immunotherapy.

Cannabis is the most used recreational drug worldwide.^9^ The functions of cannabis are mainly mediated by cannabinoids, which act on the body’s endogenous cannabinoid system (ECS), a complex network consisting of endocannabinoids, cannabinoid receptors, and enzymes catalyzing the formation and degradation of endocannabinoids.^10^ Two cannabinoid receptors have been identified in the ECS, cannabinoid receptor 1 (CNR1) and cannabinoid receptor 2 (CNR2). CNR1 is mainly expressed in the central nervous system^11^ whereas the expression of CNR2 is limited to the immune system.^12^ Consistent with the expression patterns of these two cannabinoid receptors, the ECS regulates both psychoactivity and immune functions.

Owing to its psychoactive and anti-inflammatory properties, cannabis is also used as a medicinal drug in many diseases, including cancer. THC, one of the cannabinoids identified in cannabis, has been broadly studied for medicinal use. Two THC drugs, nabilone, and dronabinol, have been approved to treat chemotherapy-caused nausea and vomiting in cancer patients.^13^ In addition to the psychoactive function, THC has been shown to suppress chronic inflammation^14^ and inhibit the growth of tumor cells^15,16^ as well. A recent study observed poor response to immunotherapy in cancer patients used cannabis,^17^ suggesting that components of cannabis might suppress antitumor immunity. Given that the combination of chemotherapy and ICB has become a widely used strategy for cancer treatment,^6^ it is important to understand whether THC interferes with immunotherapy.

In this study, using mouse models, we found that both cannabis-derived THC and the endocannabinoid AEA decreased the efficacy of PD-1 blockade by suppressing T-cell-mediated antitumor immunity. High levels of AEA in the sera indicated poor survival in cancer patients. We further discovered that CNR2 mediated the suppressive effects of cannabinoids by inhibiting the function of tumor-specific T cells. *Cnr2* deficiency greatly enhanced the antitumor activity of T cells. These results indicated a suppressive role of the ECS in antitumor immune response. To understand how CNR2 regulates T-cell function, we tagged the *Cnr2* gene with FLAG in a knock-in mouse model. The immunoprecipitation experiments and gene expression data demonstrated that CNR2 bonded to JAK1 and inhibited its downstream STAT signaling, a classic pathway regulating T-cell activation by inducing the expression of cytokines and growth factors. We thus illustrated a new mechanism of CNR2 in the suppression of T-cell activity. Overall, our results revealed that the ECS suppressed T-cell-mediated antitumor immunity through the inhibition of the JAK1-STATs signaling in T cells. Therefore, cannabis and drugs containing cannabinoids should be avoided during immunotherapy.

## Results

### THC suppresses T-cell immunity against cancer

Since cannabis has anti-inflammation properties, and THC is the key ingredient of cannabis,^18^ we hypothesized that it might affect the therapeutic efficacy of immunotherapy. Consistent with previous reports,^19^ we found that THC could inhibit the growth of tumor cells highly expressing CNR2 (Supplementary Fig. S1a, b). To avoid the effect of THC on tumor cells, we used tumor cell lines expressing low levels of CNR2 for the following experiments.

**Fig. 1:**
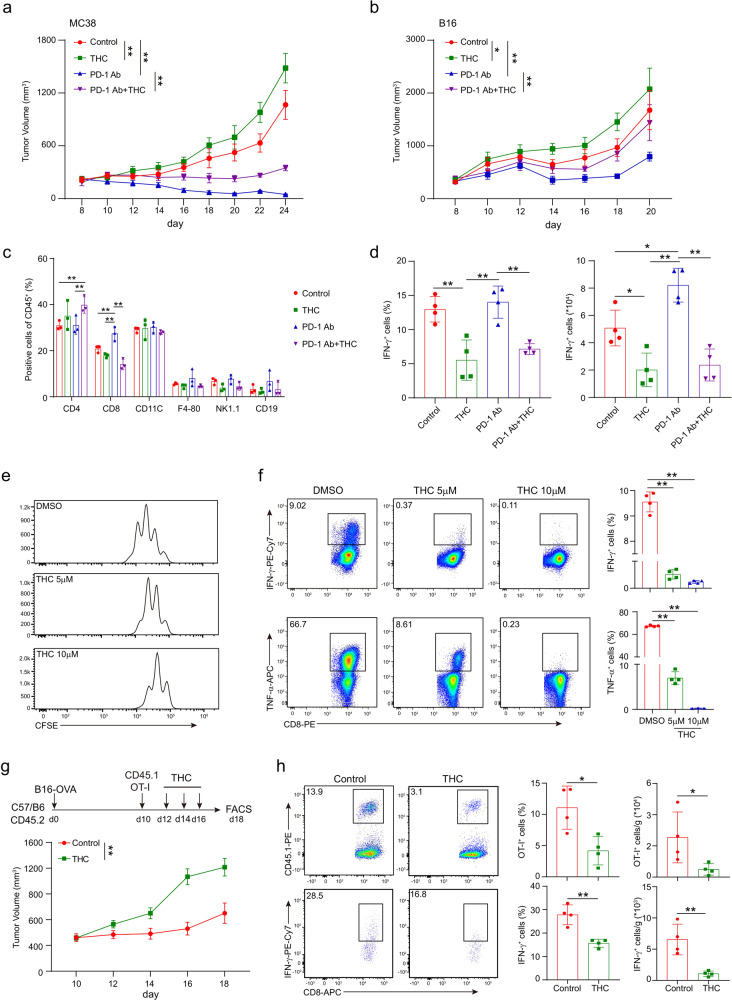
THC suppresses T-cell immunity against cancer. Mice bearing MC38 (**a**) or B16 (**b**) tumors were treated with DMSO, THC, PD-1 antibody, or THC plus PD-1 antibody on day 10 after tumor inoculation. Tumor volumes were measured every other day (two-way ANOVA, mean ± SEM; *P < 0.05, and **P < 0.01). **c** The percentages of CD3^+^ T cells, CD4^+^ T cells and CD8^+^ T cells were analyzed by flow cytometry in the B16 tumors on day 17. **d** The expression of INF-γ in T cells isolated from tumor was detected by intracellular staining after in vitro re-stimulation with PMA and Ionomycin for 4 h. Statistical analysis was performed on biological replicates, ordinary one-way ANOVA, mean ± SD; *P < 0.05, and **P < 0.01. **e**, **f** Wild-type CD8^+^ T cells were pretreated with different concentrations of THC and anti-CD3 (5 μg/ml) plus anti-CD28 (5 μg/ml) simultaneously for 48 h. Proliferation was determined by CFSE dilution assay (**e**) and the production of IFN-γ and TNF-α was detected by intracellular staining (**f**). one-way ANOVA, mean ± SD. **P < 0.01. Data are representative of three independent experiments. **g**, **h** 6–10 weeks old C57BL/6J mice were subcutaneously engrafted with 10^5^ B16-OVA tumor cells in 200 μl PBS. 10 days later, 1 × 10^6^ OT-I (CD45.1^+^) T cells were transferred intravenously through tail veins, and THC was intraperitoneally injected on day 12, 14 and 16. Tumor growth was measured every other day (**g**), and the frequencies and numbers of OT-I T cells and the production of IFN-γ in OT-I T cells after in vitro activation were measured by flow cytometry (**h**). Two-tailed unpaired Student’s t-test, mean ± SD; *P < 0.05, **P < 0.01. Data are representative of three independent experiments.

Mice bearing MC38 colon carcinoma or B16 melanoma were treated with PD-1 antibody, THC, or the combination of these two drugs. Tumor growth was measured every other day. Similar to other reports,^20^ MC38 tumors demonstrated a better response to PD-1 blockade than B16 tumors (Fig. 1a, b). Compared to DMSO controls, THC treatment significantly accelerated tumor growth in these two tumor models. Moreover, the therapeutic effect of PD-1 antibody was dramatically reduced in the combination groups, suggesting that the administration of THC might impair the antitumor immune response triggered by the PD-1 blockade. We then analyzed T-cell immune response in these groups. Consistent with the significant therapeutic effect in the PD-1 antibody group, an increased ratio of CD8^+^ T cells was observed in these tumors (Fig. 1c). However, the combination with THC diminished the effect of PD-1 blockade on both CD4^+^ T and CD8^+^ T cells while leading to a significant decrease of CD8^+^ T cells. The activity of tumor-infiltrating T cells was evaluated by in vitro activation using PMA plus Ionomycin for 4 h. While the highest production of IFN-γ was observed in T cells from tumors treated with PD-1 antibody, a significant reduction in the combination group (Fig. 1d). These data indicated that THC suppressed T-cell-mediated antitumor immunity decrease the effect of PD-1 blockade. Moreover, the therapeutic effect of PD-1 blockade was still suppressed by THC in mice depleted macrophages or B cells (Supplementary Fig. S1c, d), suggesting that THC mainly impaired T-cell immunity against cancer.

We further examined the impact of THC on T-cell activation in vitro. CD8^+^ T cells isolated from wild-type C57BL/6J mice were pretreated with THC for 12 h in medium and then activated with CD3/CD28 antibodies for 48 h. Consistent with the previous study, we found that THC significantly inhibited the proliferation of CD8^+^ T cells, as detected by the CFSE dilution assay (Fig. 1e). Reduced expression of TNF-α and IFN-γ was also observed in T cells pretreated with THC (Fig. 1f). These data showed that THC inhibited both proliferation and function of T cells during activation in vitro.

Next, we studied the effect of THC on tumor-specific T cells by using the OT-I/B16-OVA mouse model, in which OT-I T cells specifically recognize the surrogate tumor antigen ovalbumin (OVA) expressed in B16 melanoma cells. CD45.2^+^ C57BL/6J mice bearing B16-OVA melanoma were adoptively transferred with CD45.1^+^ OT-I T cells via tail vein injection and then treated with THC or DMSO as control. Similar to its effect in the PD-1 antibody treatment, administration of THC significantly diminished the therapeutic effect of the adaptively transferred OT-I T cells (Fig. 1g). A dramatic reduction in the number and function of OT-I T cells was observed in THC-treated tumors (Fig. 1h). These data further demonstrated that THC suppressed T-cell immunity against cancer.

### Endocannabinoid AEA inhibits function and proliferation of CD8^+^ T cells

**Fig. 2:**
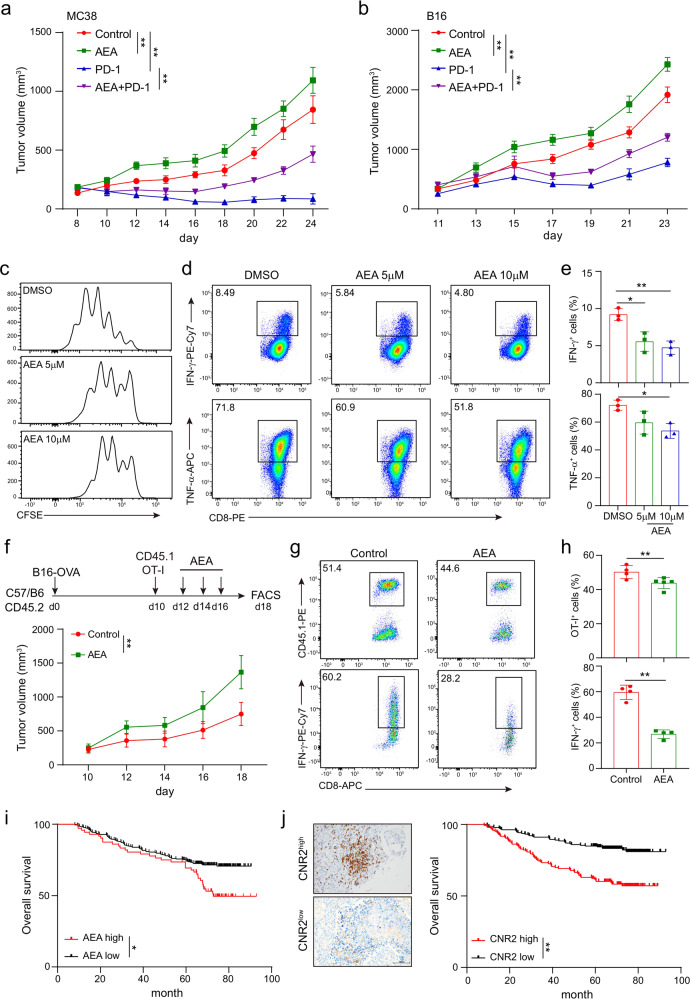
Endocannabinoid AEA inhibits function and expansion of CD8^+^ T cells. Mice bearing MC38 (**a**) or B16 (**b**) tumors were treated with DMSO, AEA, PD-1 antibody, or AEA plus PD-1 antibody on day 10 after tumor inoculation. Tumor volumes were measured every other day (two-way ANOVA, mean ± SEM, *P < 0.05, and **P < 0.01). Wild-type CD8^+^ T cells were stimulated with 5 μg/ml plate-bound anti-CD3 and anti-CD28 antibodies, and were incubated with different concentrations of endocannabinoid AEA simultaneously for 48 h. **c** The proliferation of CD8^+^ T cells was measured by CFSE dilution. **d**, **e** The production of IFN-γ and TNF-α cytokines in CD8^+^ T cells were detected by intracellular staining (mean ± SD, *P < 0.05, **P < 0.01). Statistical significance was assessed by ordinary one-way ANOVA. Data are representative of three independent experiments. B16-OVA tumors were established subcutaneously in 6–10 weeks old C57BL/6J mice 10 days before adoptive cell transfer of 1× 10^6^ OT-I T cells (CD45.1^+^) and AEA was intraperitoneally injected on day 12, 14, and 16. **f** Tumors were measured every 2 days and the volume was calculated. Data in bar graphs represent mean ± SEM, three independent experiments were performed. **g**, **h** The frequencies and numbers of OT-I T cells in tumors and the production of IFN-γ in OT-I T cells from tumor after in vitro activation with PMA and Ionomycin were shown. **i** Kaplan–Meier estimates of overall survival comparing high to low levels of AEA in serum of lung cancer patients measured by ELISA. Data are shown as mean ± SEM; log-rank test. **j** Representative IHC images of CNR2^high^ and CNR2^low^ tumor sections stained with CNR2 (left). Scale bars correspond to 100 μm. Kaplan–Meier estimates of patients’ overall survival comparing high to low expression of CNR2 (right). Statistical significance was assessed by the log-rank (Mantel–Cox) test of survival curve.

ECS has been shown to be involved in the control of chronic inflammatory injury^21,22^ suggesting that it may suppress T-cell immunity as well. Indeed, we found that AEA (an endocannabinoid) treatment significantly promoted tumor growth and diminished the therapeutic effect of PD-1 antibody both in MC38 (Fig. 2a) and B16 (Fig. 2b) mouse models. CD8^+^ T cells pretreated with AEA also showed decreased proliferation and reduced production of IFN-γ and TNF-α during in vitro activation by CD3/CD28 antibodies (Fig. 2c–e). We further investigated the impact of AEA on T-cell-mediated antitumor immunity by using the OT-I/B16-OVA model. CD45.1^+^ OT-I T cells were intravenously injected into CD45.2^+^ mice bearing B16-OVA tumors of similar size and then treated with AEA or DMSO as control. Similar to the results of the THC treatment above, significantly accelerated tumor growth was observed in mice treated with AEA (Fig. 2f). Dramatic reduction in the percentage and activities of OT-I T cells were also found in AEA-treated tumors (Fig. 2g, h). These data indicated that both cannabis-derived and endogenous cannabinoids could suppress T-cell-mediated antitumor immunity.

Having found that AEA could impair the antitumor immunity, we further checked if the AEA levels would affect disease progression in cancer patients. The AEA levels in the sera of 170 lung cancer patients were measured by ELISA. The patients were divided into high and low groups by the median level of AEA. Compared to the low-level group, patients with high levels of AEA showed worse overall survival (Fig. 2i, Table 1). We then examined if the expression of CNR2, a receptor of AEA, also affects disease progression in these patients. The CNR2 expression was measured by IHC (Fig. 2j, left). We found that high expression of CNR2 was associated with worse overall survival in these patients (Fig. 2j, right). These data suggested that the ECS could promote tumor progression through the inhibition of antitumor immunity.

**Table 1:** Clinical characteristics for the SYSUCC lung cancer cohort

	Total (*n* = 170)	AEA low (*n* = 98)	AEA high (*n* = 72)	*P*
Gender				0.9999
Male	119	67	50	
Female	51	31	22	
Age, y				0.6384
≥60	97	54	43	
<60	73	44	29	

### Cannabinoids impair T-cell-mediated antitumor immunity through CNR2

**Fig. 3:**
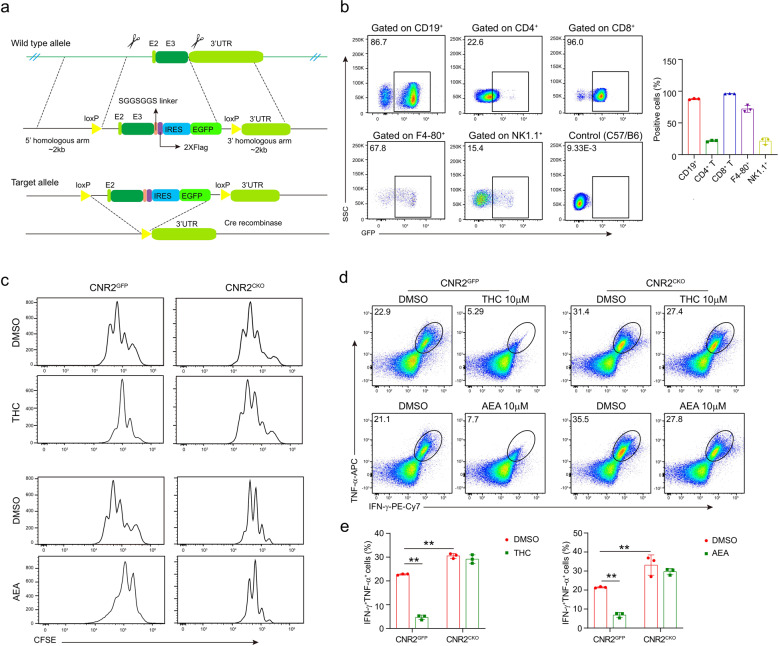
Cannabinoids impair T-cell-mediated antitumor immunity through CNR2. **a** Schematic diagram depicting the strategy used to generate *Cnr2* condition knockout (*Cnr2*^*CKO*^, CNR2^flox/flox^CD4^cre^) mice (E: exon). LoxP sites flanking exon 2 of *Cnr2* are indicated. *Cnr2-*2x*Flag-*IRES*-Egfp*^*flox/flox*^ mice (*Cnr2*^*GFP*^) were crossed with CD4^cre^ mice to delete the second exon of *Cnr2*. **b** Flow cytometry analysis of CNR2 expression from CNR2-GFP reporter mice in immune cell subsets, showing B cell (CD45^+^CD3^−^CD19^+^), T-cell (CD45^+^CD3^+^CD4^+^/CD8^+^), macrophage (CD45^+^CD3^-^F4-80^+^) and NK cell (CD45^+^CD3^−^NK1.1^+^). Cells from wild-type C57BL/6J mice were served as the negative control. *Cnr2*^*GFP*^ and *Cnr2*^*CKO*^ CD8^+^ T cells were treated with AEA or THC and stimulated by anti-CD3 plus anti-CD28 for 48 h. DMSO was used as the negative control. **c** Proliferation of CD8^+^ T cells was determined by CFSE dilution assay. **d**, **e** The production of IFN-γ and TNF-α in *Cnr2*^*GFP*^ and *Cnr2*^*CKO*^ CD8^+^ T cells were measured by intracellular staining (two-way ANOVA, mean ± SD, **P < 0.01).

CNR2 is thought to be the receptor mediating the immune function of ECS due to its primary expression in immune cells.^23^ Moreover, THC and AEA are selective agonists of CNR2.^24^ We suspected that these two cannabinoids suppress the antitumor immunity through CNR2. To investigate the role of CNR2 in the antitumor immune response, we generated a *Cnr2*-2×*Flag*-IRES-*Egfp*^*flox/flox*^ knock-in mouse line that expresses FLAG-tagged *Cnr2* with an EGFP reporter, and the second exon of the *Cnr2* gene was floxed (termed as *Cnr2*^*GFP*^). These mice were crossed with *CD4*^*Cre*^ mice to generate mice with conditional knockout of *Cnr2* in T cells (termed as *Cnr2*^CKO^, Fig. 3a, and Supplementary Fig. S2) and *Cnr2*^*GFP*^ mice were served as littermate control.

We first checked the expression of CNR2 by the EGFP reporter in different lineages of immune cells. As shown in Fig. 3B, majority of the CD8^+^ T cells and B cells expressed high levels of CNR2 while low expression was observed in CD4^+^ T cells and NK cells. Macrophages had variable levels of CNR2.

We then examined if *Cnr2* deficiency affected T-cell development. Compared to the *Cnr2*^*GFP*^ controls, increased percentages and numbers of CD4 and CD8 single-positive subsets were observed in the thymus of *Cnr2*^*CKO*^ mice, while the double-positive cells were slightly decreased. Similar phenotypes were observed in the spleen. In the mesenteric lymph node, only CD8^+^ T cells showed increased numbers in *Cnr2*^*CKO*^ mice. These data suggested that *Cnr2* ablation promoted T-cell development (Supplementary Fig. S3). We first checked the early development of T cells in the thymus. The numbers of DN1 (CD44^+^CD25^−^), DN2 (CD44^+^CD25^+^), and DN3 (CD44^−^CD25^+^) cells were not altered while DN4 cells (CD44^−^CD25^−^) were slightly reduced in the thymus of *Cnr2*^*CKO*^ mice compared to *Cnr2*^*GFP*^ mice (Supplementary Fig. S4a), which indicated that *Cnr2* was not critical for the early development of T cells in the thymus. We further examined the four distinct developmental stages defined by the expression of TCRβ and the activation marker CD69. Although the portions of TCR^-^CD69^-^ cells were slightly decreased, more TCR^+^CD69^+^ cells and TCR^+^CD69^−^ cells were observed in *Cnr2*^*CKO*^ mice compared to *Cnr2*^*GFP*^ mice (Supplementary Fig. S4a), indicating that *Cnr2* deficiency promoted the positive selection of T cells in the thymus and exported more T cells to the periphery. Expression of activation markers such as CD69 and CD25 was comparable on CD8^+^ T cells in aged *Cnr2*^*CKO*^ and *Cnr2*^*GFP*^ mice (Supplementary Fig. S4b). Histological analysis showed no inflammation in the organs of these aged mice in either group (Supplementary Fig. S4c). These data indicated that the negative selection in the thymus of *Cnr2*^*CKO*^ mice was normal.

Since increased T-cell numbers were observed in the periphery, we also examined the homeostasis of *Cnr2*^*CKO*^ and *Cnr2*^*GFP*^ T cells. *Cnr2*^*GFP*^ (CD45.1^+^CD45.2^+^) and *Cnr2*^*CKO*^ (CD45.2^+^) CD8^+^ T cells were mixed in a ratio of 1:1, and then transferred into Rag2^−/−^ recipient mice (CD45.1^+^) (Supplementary Fig. S5a). The frequencies of these two kinds of T cells in the lymph node (LN) and spleen (SP) were measured in 7 days. We found that the ratios of *Cnr2*^*CKO*^ to *Cnr2*^*GFP*^ T cells were slightly increased, which indicated that *Cnr2* ablation increased T-cell homeostasis (Supplementary Fig. S5b).

Next, we investigated if THC and AEA would suppress T-cell activation through CNR2. CD8^+^ T cells isolated from *Cnr2*^*GFP*^ or *Cnr2*^*CKO*^ mice were pretreated with THC or AEA for 12 h and then stimulated with CD3/CD28 antibodies for 48 h. Although pretreatment of THC or AEA both significantly inhibited the proliferation and function of wild-type *Cnr2*^*GFP*^ CD8^+^ T cells, these two cannabinoids did not affect the proliferation and the IFN-γ and TNF-α production of *Cnr2*^*CKO*^ CD8^+^ T cells, indicating that cannabinoids inhibited T-cell function through CNR2 (Fig. 3c–e).

Surprisingly, in the DMSO treated groups, significantly increased proliferation and higher levels of IFN-γ and TNF-α were observed in the *Cnr2*^*CKO*^ T cells, compared to *Cnr2*^*GFP*^ T cells, suggesting that CNR2 suppressed T-cell function. Similar results were observed when these T cells were activated in vitro without DMSO (Supplementary Fig. S6). These data supported that CNR2 itself could directly inhibit T-cell proliferation and function.

### *Cnr2* deficiency promotes T-cell-mediated antitumor immunity

**Fig. 4:**
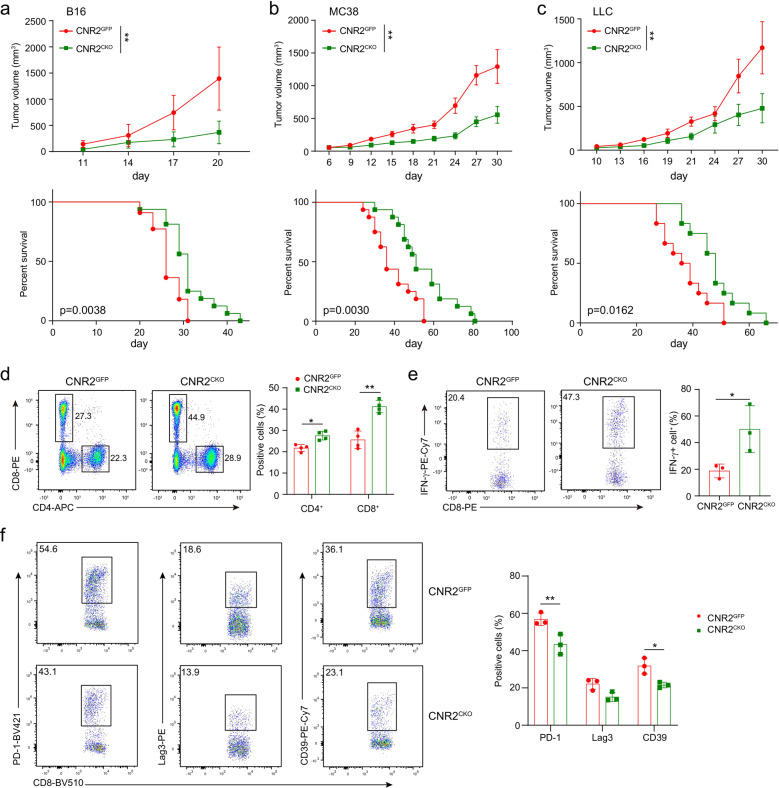
CNR2 facilitates tumor development by suppressing immune response. Tumor growth and survival were assessed in B16 (**a**), MC38 (**b**) and LLC (**c**) models in *Cnr2*^*GFP*^ and *Cnr2*^*CKO*^ mice. Data are representative of three independent experiments. Statistical significance was assessed by ordinary one-way ANOVA or log-rank (Mantel–Cox) test of survival curve, mean ± SEM. **P < 0.01. **d** Flow cytometric analysis of CD4^+^ and CD8^+^ T cells of *Cnr2*^*GFP*^ and *Cnr2*^*CKO*^ mice bearing B16 tumor. **e** The production of IFN‐γ in CD8^+^ T cells from B16 tumors was assessed by flow cytometric analysis. Data are representative of three independent experiments. Statistical significance was assessed by two-way ANOVA (**d**) or two-tailed unpaired Student’s *t*-test (**e**), mean ± SD, *P < 0.05, **P < 0.01. **f** Flow cytometry (left) and quantification (right) of PD-1, LAG3, and CD39 positive cells in *Cnr2*^*GFP*^ and *Cnr2*^*CKO*^ OT-I cells from B16-OVA tumors. Two-tailed unpaired Student’s t-test, mean ± SD, *P < 0.05, **P < 0.01.

Given that CNR2 inhibited T-cell activation in vitro, we wondered whether it would affect antitumor immunity in vivo. To this end, we employed three tumor models, MC38, B16, and LLC. Compared to wild-type mice, all of these three kinds of tumors showed slower growth and prolonged survival in *Cnr2*^*CKO*^mice (Fig. 4a–c). The frequency of CD8^+^ T cells was significantly increased in the tumors from *Cnr2*^*CKO*^ mice (Fig. 4d). Moreover, T cells isolated from tumors of *Cnr2*^*CKO*^ mice produced more IFN-γ during the activation in vitro (Fig. 4e). The expression of exhaustion markers such as PD-1, LAG3, and CD39 was also reduced in T cells from tumors of *Cnr2*^*CKO*^ mice compared to wild-type mice (Fig. 4f). These data indicated that *Cnr2* deficiency enhanced the antitumor function of T cells, thus inhibiting tumor growth.

**Fig. 5:**
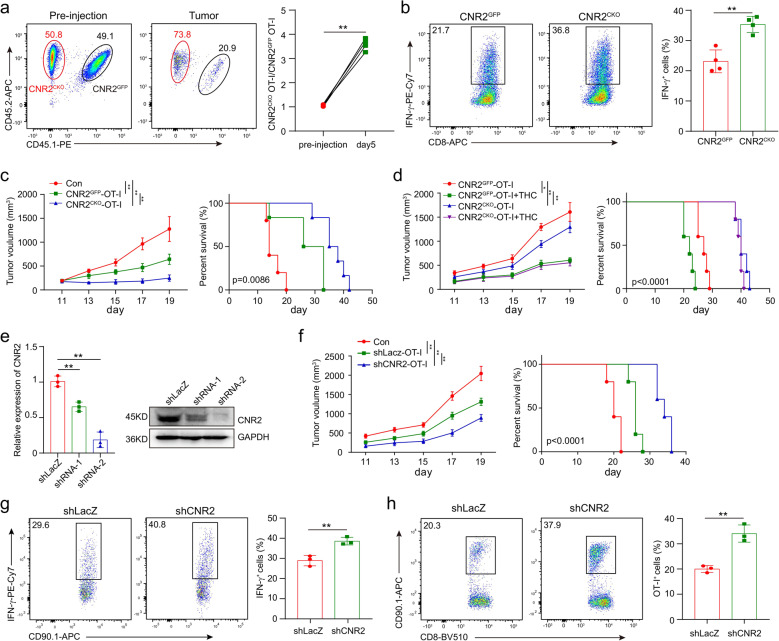
*Cnr2* deficiency promotes T-cell-mediated antitumor immunity. **a** *Cnr2*^*GFP*^ (CD45.1^+^ CD45.2^+^) and *Cnr2*^*CKO*^ (CD45.2^+^) OT-I CD8^+^ T cells were 1:1 mixed and intravenously injected into mice (CD45.1^+^) with B16-OVA tumor. Flow cytometric analysis shows the frequencies of *Cnr2*^*GFP*^ and *Cnr2*^*CKO*^ OT-I T cells from TILs in tumors (left). The representative ratio of wild-type to *Cnr2*^*CKO*^ OT-I T cells in the tumor were evaluated on day 5 (right). **b** Flow cytometry analysis of the production of IFN‐γ in *Cnr2*^*GFP*^ and *Cnr2*^*CKO*^ OT-I T cells isolated from tumors stimulated with PMA and Ionomycin in vitro. Data are representative of three independent experiments. Statistical significance was assessed by two-tailed unpaired Student’s t-test, mean ± SD. *P < 0.05 and **P < 0.01. **c** Mice bearing B16-OVA tumors were treated with 1 × 10^6^ *Cnr2*^*GFP*^ or *Cnr2*^*CKO*^ OT-I T cells. PBS was used as control. Tumor volume was measured every other day (mean ± SEM, **P < 0.01) (Left). The survival curves of the three groups were compared (right). Statistical significance was assessed by the two-way ANOVA (left), or log-rank (Mantel–Cox) test of survival curve (right). **d** Mice bearing B16-OVA tumors were treated with 1 × 10^6^ *Cnr2*^*GFP*^, *Cnr2*^*CKO*^ OT-I T cells, *Cnr2*^*GFP*^ OT-I T cells plus THC and *Cnr2*^*CKO*^ OT-I T cells plus THC. Tumor volume were measured every other day (mean ± SEM, **P < 0.01) (left). The survival curves of the three groups were compared (right). Statistical significance was assessed by the two-way ANOVA (left), or log-rank (Mantel–Cox) test of survival curve (right). **e** T cells were transduced for 48 h by lentivirus and positive cells were sorted by flow cytometry. The expression of CNR2 was validated by qPCR and Western blotting. **f** Mice bearing B16-OVA tumors were treated with 1 × 10^6^ *shLacZ* or *shCnr2* OT-I T cells. PBS was used as control. Tumor volume was measured every other day (mean ± SEM, **P < 0.01). The survival curves of the three groups were compared. Statistical significance was assessed by the two-way ANOVA, or log-rank (Mantel–Cox) test of survival curve. **g** The frequencies of OT-I T cells in tumors were shown. **h** Flow cytometry analysis of the production of IFN-γ in *shLacZ* and *shCnr2* OT-I T cells isolated from tumors stimulated with PMA and Ionomycin in vitro. Statistical significance was assessed by two-tailed unpaired Student’s *t*-test, mean ± SD, **P < 0.01.

We then examined the effect of CNR2 on tumor-specific T cells in the OT-I/B16-OVA model. *Cnr2*^*GFP*^ OT-I (CD45.1^+^CD45.2^+^) and *Cnr2*^*CKO*^ OT-I (CD45.2^+^) T cells were mixed in a ratio of 1:1, and then adoptively transferred into C57BL/6J recipient mice (CD45.1^+^) with established subcutaneous B16-OVA tumors (Fig. 5a). The frequencies of these two kinds of OT-I T cells in B16-OVA tumors were measured 5 days after transfer. More *Cnr2*^*CKO*^ OT-I cells were found in the tumors of recipient mice. The ratio of *Cnr2*^*CKO*^ to *Cnr2*^*GFP*^ OT-I cells was also significantly increased (Fig. 5a). In addition, *Cnr2*-deficient OT-I T cells produced more IFN-γ than their *Cnr2*^*GFP*^ counterparts (Fig. 5b). These data demonstrated that *Cnr2* deficiency in tumor-specific T cells enhanced their expansion and function in the tumor microenvironment.

Having found that *Cnr2* deficiency enhanced the activities of tumor-specific T cells, we questioned whether these T cells could have superior therapeutic efficacy than wild-type T cells in tumor treatment. Mice bearing B16-OVA tumors were treated with *Cnr2*^*CKO*^ or *Cnr2*^*GFP*^ OT-I T cells through adoptive transfer. As expected, mice treated with *Cnr2*^*CKO*^ OT-I T cells showed much slower tumor growth and longer survival than the *Cnr2*^*GFP*^ OT-I T-cell-treated group (Fig. 5c). Moreover, THC administration impaired the efficacy of the *Cnr2*^*GFP*^ OT-I cells but did not affect the efficacy of the *Cnr2*^*CKO*^ OT-I T cells in treating B16-OVA tumors (Fig. 5d). These data suggested that targeting *Cnr2* in T cells could be a potential approach to improve the efficacy of T-cell adoptive transfer therapy.

Since *Cnr2* deficiency slightly affects T-cell development, we further used shRNA to knockdown *Cnr2* in WT OT-I T cells, and then examined their function in B16-OVA tumors. Both qPCR and Western blotting showed that the *Cnr2* shRNA-2 efficiently knocked down the expression of CNR2 in T cells (Fig. 5e). Mice bearing B16-OVA tumors were then treated with OT-I T cells traduced with *Cnr2* shRNA-2 or control LacZ shRNA. Similar to *Cnr2*-deficient T cells, *Cnr2* knockdown OT-I T cells significantly inhibited the growth of B16-OVA tumors and improved survival of tumor-bearing mice compared to the OT-I T cells transduced with LacZ shRNA (Fig. 5f). In addition, *Cnr2* knockdown increased OT-I T cell numbers in tumors (Fig. 5g), and enhance the expression of IFN-γ in these T cells (Fig. 5h). These data indicated that *Cnr2* knockdown enhanced the function of tumor-specific T cells in the tumor microenvironment.

Taken together, our results revealed that the ECS attenuated T-cell-mediated antitumor immunity through CNR2.

### CNR2 binds to JAK1 and inhibits the STATs signaling

**Fig. 6:**
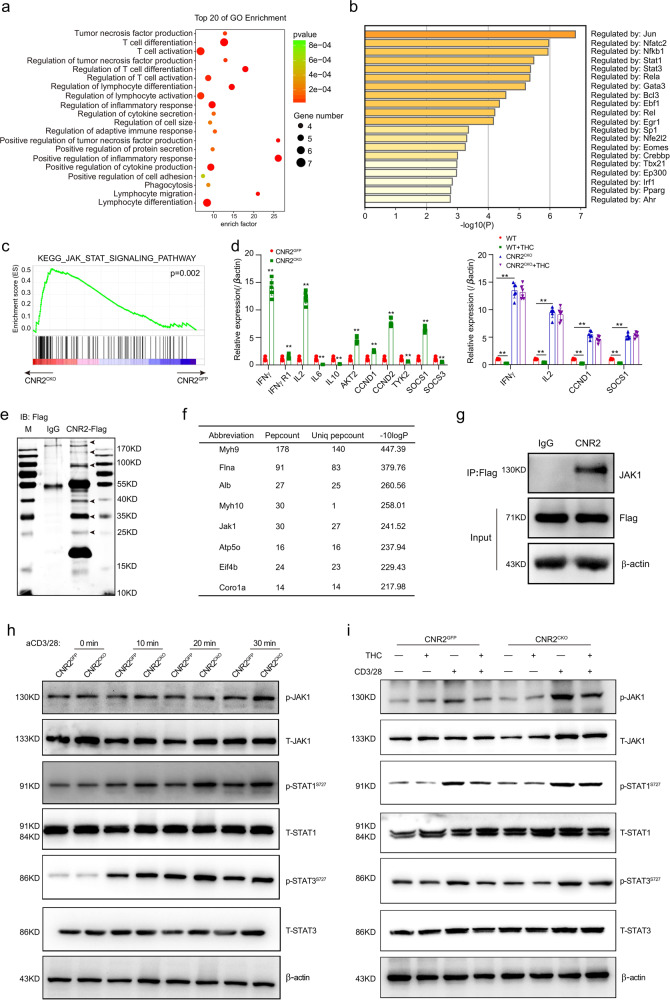
CNR2 binds to JAK1 and inhibits the STATs signaling. CNR2 binds to JAK1 and inhibits the STATs signaling. **a**, **b** Gene Ontology (GO) category analysis and Heatmap GO analysis of RNA-seq data of *Cnr2*^*CKO*^ and *Cnr2*^*GFP*^ CD8^+^ T cells treated with anti-CD3 plus (5 μg/ml) anti-CD28 (5 μg/ml) for 24 h by using Metascape website (http://metascape.org). **c** GSEA analysis of the differentially expressed genes (RNA-seq datasets) of the JAK-STAT signaling pathway in *Cnr2*-deficient CD8^+^ T cells versus wild-type CD8^+^ T cells. **d** qPCR validation of the expression of genes downstream JAK-STAT signaling pathway in *Cnr2*-deficient and wild-type CD8^+^ T cells (Left), and in THC-treated *Cnr2*-deficient and wild-type CD8^+^ T cells (Right). Data are presented as the mean ± SD. of three biological replicates. **P < 0.01. **e** Mass spectrum analysis of CNR2 associated proteins in CD8^+^ T cells from *Cnr2-*2x*Flag-*IRES*-Egfp*^*flox/flox*^ reporter mice after Flag pull-down assay. Six protein bands detected by silver-staining in the FLAG group but not in the IgG group were cut and performed mass spectrum analysis. **f** The top ten identified peptides were shown in the list. **g** *Cnr2-*2x*Flag-*IRES*-Egfp*^*flox/flox*^ CD8^+^ T cells were treated with THC or DMSO as a control for 24 h. Cell lysates were then immunoprecipitated with Flag antibody and analyzed by immunoblot with anti-JAK1 and anti-Flag. **h** *Cnr2*^*GFP*^ and *Cnr2*^*CKO*^ CD8^+^ T cells were stimulated with anti-CD3 plus anti-CD28 for 10–30 min. Cell lysates were analyzed by immunoblot with anti-phosphorylated JAK1, anti-phosphorylated STAT1, anti-phosphorylated STAT3, anti-total STAT1, and anti-total STAT3. **i** *Cnr2*^*GFP*^ and *Cnr2*^*CKO*^ CD8^+^ T cells were pretreated with THC or DMSO for 24 h and then stimulated by anti-CD3 plus anti-CD28 for 30 min. Cell lysates were analyzed by immunoblot with anti-phosphorylated JAK1, anti-phosphorylated STAT1, anti-phosphorylated STAT3, anti-total STAT1, and anti-total STAT3.

Finally, we addressed how CNR2 impaired T-cell activities. To identify the signaling pathways regulated by CNR2 in T cells, we performed an RNA-seq of *Cnr2*^*CKO*^ and *Cnr2*^*GFP*^ CD8^+^ T cells activated by anti-CD3/28 antibody for 24 h. Gene Ontology (GO) category analysis showed that T-cell differentiation and activation were the two most differentiated biological processes (Fig. 6a), consistent with the enhanced proliferation and effector function of *Cnr2*-deficient T cells. We further performed the Heatmap GO analysis on the Metascape website (http://metascape.org) to find out the key regulators. Interestingly, we found that two of the mainly affected genes were *Stat1* and *Stat3* (Fig. 6b). Gene Set Enrichment Analysis (GSEA) further indicated a strong activation of the JAK-STAT signaling pathway in *Cnr2*-deficient T cells (Fig. 6c). Additionally, the target genes of the JAK-STAT signaling including *Ifnγ*, *Ifngr1*, *Il2*, *Akt2*, *Ccnd1*, *Ccnd2*, and *Socs1* were upregulated and *Il6*, *Il10*, *or Socs3* were downregulated, validating the activation of the JAK-STAT signaling pathway in *Cnr2*-deficient T cells (Fig. 6d). Consistently, the expression of downstream genes such as *Ifnγ*, *Il2*, *Ccnd2*, and *Socs1* was also suppressed by THC in *Cnr2*^*GFP*^ T cells but not in *Cnr2*^*CKO*^ T cells (Fig. 6d).

To further explore the signaling pathways regulated by CNR2 in T cells, we employed immunoprecipitation followed by mass spectrometry to identify proteins interacting with CNR2. As mentioned above, the CNR2 protein was tagged by FLAG in the knock-in mouse line. We sorted CD8^+^GFP^+^ T cells from the spleen and lymph nodes of the knock-in mice and performed immunoprecipitation using FLAG antibody or control IgG (Fig. 6e). Differential bands were collected and mass spectrometry was performed. Consistent with the findings in RNA-seq, we found that JAK1 was one of the top proteins binding to CNR2, while the others belonged to unspecific cell adhesion proteins (Fig. 6f). We further confirmed the binding between CNR2 and JAK1 by co-immunoprecipitation assay (Fig. 6g).

Next, we compared the expression of pJAK1 or pSTAT1 and pSTAT3 in *Cnr2*^*CKO*^ and *Cnr2*^*GFP*^ control T cells during the activation in vitro. Compared to *Cnr2*^*GFP*^ T cells, increased levels of phosphorylated JAK1, STAT1, and STAT3 proteins were observed in *Cnr2*^*CKO*^ T cells in a time-dependent manner after the activation, while the levels of total JAK1 or STAT1 and STAT3 proteins remained unchanged (Fig. 6h).

We further checked if THC suppressed the JAK-STAT signaling in T cells through CNR2. *Cnr2*^*GFP*^ cells and *Cnr2*^*CKO*^ T cells were pretreated with or without THC and then activated by CD3 and CD28 antibodies for 30 min. THC treatment decreased the phosphorylation of STAT1 and STAT3 in *Cnr2*^*GFP*^ T cells but not in *Cnr2*^*CKO*^ T cells during activation (Fig. 6i). These data suggested that THC inhibited the JAK1-STAT1/3 signaling in T cells through CNR2.

Overall, our results indicated that the ECS impaired T-cell activity through the inhibition of the JAK1-STATs signaling in T cells.

## Discussion

Overall, our results revealed that both cannabis-derived and endogenous cannabinoids impaired T-cell-mediated antitumor immunity by inhibiting the JAK1-STATs signaling in T cells through CNR2. These findings indicated that the ECS is involved in the suppression of the antitumor immune response, suggesting that cannabis and drugs containing THC should be avoided during cancer immunotherapy. Cannabis is also used to treat cancer pain and increase appetite in patients with advanced disease.^25^ Cannabis-mediated suppression of antitumor immunity might expedite the disease progression. Alternative medicines are thus needed for these treatments.^26^ Given the wide use of cannabis in a number of medical conditions, its immunosuppressive effect on T cells should be considered in the treatment of these diseases. Moreover, marijuana is one of the most used illegal drugs for psychoactive recreation.^17^ In addition to its effect on the nervous system,^10^ long-term use of marijuana may result in continuous suppression on the immune system.^27–29^ Indeed, studies have shown that persistent marijuana consumption increased the incidence of diseases associated with a compromised immune function such as lung inflammation, pulmonary infection, and bronchitis.^30,31^ Therefore, the non-medical use of cannabis and cannabinoids must be highly cautious.

Recent studies have shown that corticosteroids treating the immune-relevant side effects of ICB reduced the therapeutic efficacy of ICB,^7,32^ suggesting that drugs managing side effects might impair antitumor immunity. Due to the low response rates to ICB therapy in most of the cancer types, improving the therapeutic efficacy of ICB through combination strategy has become the main direction in immunotherapy.^33^ Meanwhile, the combination therapy of ICB with other drugs could increase the incidence of advanced events, which needs more ancillary drugs to treat both the immune-relevant and the combined drug-mediated side effects.^4^ Combinations of ICB and chemotherapies have shown promising results in several clinical trials.^1^ Here, we showed that THC treating chemotherapy-caused nausea also reduced the efficacy of ICB by suppressing T-cell-mediated antitumor immunity. Our findings thus highlight the necessity of assessing the impact of ancillary drugs on immune response before using them with immunotherapy.

Studies have shown that CNR2 is mainly expressed in immune cells and mediates anti-inflammatory effects by inhibiting cytokine expression and migration of these cells.^34,35^ Although CNR2 has been shown to trigger the G_i/o_-mediated signaling pathways,^36,37^ the molecular mechanisms underlying these immunosuppressive effects remain unclear. Using CNR2 reporter mice, we found that different clusters of immune cells showed variable expression of CNR2, suggesting that the ECS might have different effects on these cells. Compared to CD4^+^ T cells, higher expression of CNR2 was observed in CD8^+^ T cells. We further showed that CNR2 binds to JAK1 and inhibits STAT signaling in T cells. Our findings, therefore, demonstrated that the ECS regulates T-cell function through the classic JAK1/STATs signaling pathway as well.

We found that *Cnr2* deficiency promoted the positive selection of thymocytes and T-cell homeostasis, which increased the numbers of CD4^+^ and CD8^+^ T cells in the thymus and periphery. Consistent with these findings, our data showed that the ECS inhibited T-cell function and proliferation by suppressing the JAK-STAT signaling through CNR2. Given that the JAK-STAT signaling is involved in the survival and proliferation of T cells, it is likely that enhanced JAK-STAT signaling resulted from *Cnr2* ablation promoted T-cell survival during positive selection and proliferation in the periphery. Interestingly, these increased T cells in the periphery did not cause autoimmunity in *Cnr2-*deficient mice, suggesting that the CNR2-mediated JAK-STAT signaling might have a limited effect on the negative selection of T cells in the thymus. Although the developmental advantage might enhance the antitumor immunity in *Cnr2*-deficient mice, we found that shRNA knockdown of *Cnr2* in wild-type OT-I T cells greatly enhanced their activities against tumor. These data suggested that the ECS mainly inhibits T-cell function and proliferation.

Consistent with the suppressive role of the ECS in antitumor immunity, we found that high levels of the endocannabinoid AEA were associated with poor survival of cancer patients. These data suggest that targeting the ECS might be a potent therapy for cancer treatment. Nevertheless, targeting the ECS might lead to psychiatric side effects due to its function on the nervous system. Since CNR2 is mainly expressed in immune cells, specific blockade of the CNR2 signaling may avoid the neurological side effects, and thus offer a promising option for the treatment of cancer and other immune-related diseases.

## Materials and methods

### Cell lines, antibodies, and reagents

The mouse cancer cell lines B16 and LLC were purchased from ATCC (Manassas, VA). B16-OVA and MC38 cells were generously provided by Dr. Pan Zheng (University of Maryland; Baltimore, MD). All cell lines are mycoplasma free. The cells were maintained in Dulbecco’s modified Eagle’s medium or Roswell Park Memorial Institute 1640 medium (RPMI-1640) supplemented with 10% FBS, 1% penicillin-streptomycin, 2mM L-glutamine, and 1 mM sodium pyruvate at 37 °C in 5% CO_2_.

For western blot, the anti-CNR2 antibody was purchased from Invitrogen (CA, USA). Anti-pJAK1-Tyr1034/1035, anti-pSTAT1-Ser727 antibodies, anti-STAT1, anti-pSTAT3-Ser727 antibodies, anti-STAT3, anti-Flag, and secondary antibodies HRP-mouse or HRP-rabbit were purchased from Cell Signaling Technology (Boston, Massachusetts USA). Anti-β-actin, anti-JAK1 were purchased from Proteintech (Chicago, IL, USA).

AEA and THC were purchased from Sigma (Sigma‐Aldrich, CA, USA). The anti-mouse PD-1 antibody was purified from hybridoma (clone G4; kindly provided by Dr. Lieping Chen, Yale University, New Haven, CT) culture supernatant.

### Mice

C57BL/6J mice were purchased from Charles River Laboratory (Beijing, China). CD4-Cre and OT-I (CD45.1^+^) transgenic mice were purchased from the Jackson Laboratory (Bar Harbor, ME). The *Cnr2*-2×*Flag*-IRES-*Egfp*^*flox/flox*^ knock-in mice were generated by targeting the *Cnr2* gene locus in a C57BL/6J mouse ES line through homologous recombination and CRISPR/Cas9 technology (Beijing Biocytogen). This knock-in mouse line expresses FLAG-tagged CNR2 and the EGFP reporter. The second exon of *Cnr2* gene was floxed in this line. These mice were crossed with *CD4*^*Cre*^ mice to generate mice with conditional knockout of *Cnr2* in T cells. CD45.1^+^ *Cnr2*^*CKO*^ OT-I mice were generated by crossing *Cnr2*^*CKO*^ mice with CD45.1^+^ OT-I mice. The mice were maintained in the specific pathogen-free facility of the Sun Yat-sen University (Guangzhou, China). The animal experimental protocols were approved by the Institutional Animal Care and Use Committee of the Sun Yat-sen University Cancer Center (SYSUCC; Guangzhou, China).

### Human tumor samples

Paraffin-embedded tissue samples and matched serum of lung cancer patients were obtained at SYSUCC. All specimens were obtained with written informed consent and the experimental protocols were approved by the institutional review committee.

### Murine tumor models and treatments

1 × 10^5^ B16, B16-OVA, MC38, or LLC tumor cells resuspended in 200 µL PBS were injected subcutaneously into the mice. Tumor volumes were measured every 2 days after palpable tumors were observed. Tumor volumes were calculated by the following formula: tumor volume=0.5 × length × width.^2^ Mice were euthanized when tumor volume exceeded 2000 mm.^3^

THC and AEA were dissolved in the solution composed of ethanol:Tween 80: Saline = 1:1:18 at a concentration of 10 mg/ml. The mice were treated with 10 mg/kg THC or AEA intraperitoneally every 2 days for 2 weeks. PD-1 antibodies (200 µg) were administered intraperitoneally twice a week throughout the experiment.

For the tumor treatment by adoptive transfer of OT-I T-cell,^38^ B16-OVA cells were injected subcutaneously into 8 weeks old female C57BL/6J mice. On day 12, mice bearing tumors of similar size were divided into different groups, and intravenously received 1 × 10^6^ CD8^+^ OT-I T cells on day 12 and day 17, which were cultured with IL2, IL7, and IL15 (Peprotech, Rocky Hill, USA) for 5 days.

For the competition assay of OT-I T cells in the B16-OVA model, 12 days after tumor inoculation, cytokine-cultured wild-type and *Cnr2*^*CKO*^ OT-I T cells were mixed at a ratio of 1:1 and transferred into tumor-bearing mice (1 × 10^6^ mixed OT-I T cells/mouse). Four days later, the ratio, function and proliferation of transferred OT-I T cells in tumors were analyzed by flow cytometry.

### Tumor-infiltrating lymphocytes flow cytometry and cell sorting

Tumor tissues were cut into small pieces and washed with PBS containing 2% FBS. The tumors were digested in 15 ml RPMI supplemented with 2% FBS, 50 U/ml Collagenase Type IV (Invitrogen, California, USA), 20 U/ml DNase (Roche, Indianapolis, IN) and incubated at 37 °C for 2 h while gently shaking and further processed with the gentleMACS dissociator (Miltenyi Biotech, Bergisch Gladbach, Germany). Digested tumors were then filtered through a 70 µm strainer after washed three times with PBS. Spleens and draining lymph nodes were mechanically dissociated with gentleMACS dissociator in RPMI-1640 medium supplemented with 2% FBS. Dissociated spleens were passed through a 70 µm strainer and washed three times with PBS. Red blood cells were lysed after 10 min of incubation with ammonium chloride solution and then washed with PBS containing 2% FBS. Cells were resuspended in PBS with 2% FBS, then specific antibodies were added and staining was continued for 30 min on ice. For intracellular cytokine staining, cells were stimulated with Leukocyte Activation Cocktail (BD Biosciences, 550583). After surface markers staining, the cells were fixed and permeabilized according to the manufacturer’s instructions. All flow cytometric data were collected on BD Fortessa X20 (BD Biosciences, San Jose, CA) and performed using FlowJo analysis software v10.4.

For cell sorting of GFP^+^ from *Cnr2*^*GFP*^ reporter mice, spleens and lymph nodes were collected and then passed through a 70 µm strainer. Negative selection was carried out with a negative CD8 isolation kit following the manufacturer’s instructions (Biolegend, San Diego, CA). After a washing step, CD8^+^GFP^+^ T cells were then sorted by BD FACSAria II (BD Biosciences, San Jose, CA).

### B cell depletion in mouse model

Each C57BL/6J mouse bearing B16-OVA tumors was injected i.v. with 250 µg of Ultra-LEAF™ purified mAb SA271G2 or PBS (negative control). To study the presence of B cells, blood was collected and stained with CD20 antibody.

### Macrophage depletion in mouse model

Macrophages were depleted by i.v. application of clodronate liposomes (200 μl per mouse; YEASEN, Shanghai, China). The efficiency of macrophages depletion was evaluated using F4-80 antibody by flow cytometry.

### T-cell isolation and activation in vitro

Mouse CD8^+^ T cells were isolated from the spleens and lymph nodes using negative selection kits and then activated with plate-bound 5 µg/ml CD3 and 5 µg/ml CD28 antibodies (Invitrogen, California, USA). OT-I T cells were activated with 1 µg/ml OVA_257-264_ (SIINFEKL) peptides. CFSE dilution and intracellular staining were performed on day 2.

### Retroviral transduction of mouse T cells

shRNAs were cloned into the pMSCV-IRES-GFP-HA retroviral plasmid with the miR30 backbone. 293T cells were transfected with the retroviral plasmid and pCL-Eco helper plasmid by PEI regent. The retrovirus supernatant was harvested 48 h after transfection and filtered by 0.45-mm strainer (Millipore, MA USA). Before infection, T cells were activated by CD3-28 antibody for 2 days. Then 2 × 10^5^ T cells were transduced with 1 ml retrovirus supernatant plus 4 μg/ml polybrene in a retronectin coated 24-well plate, then centrifuged at 2000 × *g* for 1 h at room temperature and incubated for another 48 h, and then replaced with fresh T cells medium supplemented with 20 ng/ml rhIL2. Two different shRNAs were tested, and studies were performed using the one with best knockdown of protein expression. The shRNA sequences (5′–3′):

shLacZ:

F:CCCCTGCCCGTCAGTATCGGCGGAATTCCTGTGACATGTCAAAAAGAATTCCGCCGATACTGACGGGCT;

R:CACCAGCCCGTCAGTATCGGCGGAATTCTTTTTGACATGTCACAGGAATTCCGCCGATACTGACGGGCA.

shCNR2-1:

F:CCCCTCTATGGTCAATCCTATCATTTACCTGTGACATGTCAAAAAGTAAATGATAGGATTGACCATAGT;

R:CACCACTATGGTCAATCCTATCATTTACTTTTTGACATGTCACAGGTAAATGATAGGATTGACCATAGA.

shCNR2-2:

F:CCCCTTGCCTTGTTAACTCTATGGTCAACTGTGACATGTCAAAAATTGACCATAGAGTTAACAAGGCAT;

R:CACCATGCCTTGTTAACTCTATGGTCAATTTTTGACATGTCACAGTTGACCATAGAGTTAACAAGGCAA.

### RNA isolation and real-time quantitative PCR (qRT-PCR)

Total RNA was isolated from T cells using the RNeasy mini kit (Invitrogen, California, USA) according to the manufacturer’s protocol. The quantity and quality of RNA were determined by Nanodrop. Reverse transcription was performed using cDNA Synthesis Kit (TransGen Biotech, Beijing, China) and quantitative real-time PCR was carried out using Bio-Rad SYBR green master mix (TransGen Biotech, Beijing, China) and reactions were run on an Applied Biosystems. The expression of individual genes was normalized to β-actin expression on the base of the ∆Ct method.

Primers:

mIFN-γ forward: 5′-CAGCAACAGCAAGGCGAAAAAGG-3′

mIFN-γ reverse: 5′-TTTCCGCTTCCTGAGGCTGGAT-3′

mIFNγR1 forward: 5′-CTTGAACCCTGTCGTATGCTGG-3′

mIFNγR1 reverse: 5′-TTGGTGCAGGAATCAGTCCAGG-3′

mCCND1 forward: 5′-GCAGAAGGAGATTGTGCCATCC-3′

mCCND1 reverse: 5′-AGGAAGCGGTCCAGGTAGTTCA-3′

mCCND2 forward: 5′-GCAGAAGGACATCCAACCGTAC-3′

mCCND2 reverse: 5′-ACTCCAGCCAAGAAACGGTCCA-3′

mIL2 forward: 5′-GCGGCATGTTCTGGATTTGACTC-3′

mIL2 reverse: 5′-CCACCACAGTTGCTGACTCATC-3′

mIL6 forward: 5′-TACCACTTCACAAGTCGGAGGC-3′

mIL6 reverse: 5′-CTGCAAGTGCATCATCGTTGTTC-3′

mIL10 forward: 5′-CGGGAAGACAATAACTGCACCC-3′

mIL10 reverse: 5′-CGGTTAGCAGTATGTTGTCCAGC-3′

mAKT2 forward: 5′-CCAACACCTTTGTCATACGCTGC-3′

mAKT2 reverse: 5′-GCTTCAGACTGTTGGCGACCAT-3′

mSOCS1 forward: 5′-AGTCGCCAACGGAACTGCTTCT-3′

mSOCS1 reverse: 5′-GTAGTGCTCCAGCAGCTCGAAA-3′

mSOCS3 forward: 5′-GGACCAAGAACCTACGCATCCA-3′

mSOCS3 reverse: 5′-CACCAGCTTGAGTACACAGTCG-3′

mTYK2 forward: 5′-GCTTTCCTGCATGGTGTTTGCG-3′

mTYK2 reverse: 5′-TGTCGCCGTAACCACACATCCA-3′

β-actin forward: 5′-CATTGCTGACAGGATGCAGAAGG-3′

β-actin reverse: 5′-TGCTGGAAGGTGGACAGTGAGG-3′

### Protein extraction and western blotting analysis

Cells were washed with PBS and RIPA lysis buffer (Beyotime, Shanghai, China) contained with protease inhibitor, and then lysed on ice with phosphatase inhibitor (Roche, Basel, Switzerland) for 15 min before cleared by 15,000 rpm at 4 °C. Protein concentrations were measured by BCA protein assay, adjusted equally between samples, and boiled at 95 °C for 5 min after adding SDS loading buffer (5×). Equal volume and equal quantity of protein samples were subjected to SDS-PAGE gel and transferred to a PVDF membrane (Millipore, MA, USA). The membranes were blocked in 5% skim milk at room temperature for 1 h and incubated with primary antibodies at 4 °C overnight with gently shaking. On the next day, the membranes were washed with TBST [10 mM Tris-HCl, pH 8.0, 150 mM NaCl, 0.1% (v/v) Tween-20] and incubated with secondary HRP antibodies in TBST at room temperature for 1 h. ECL (Millipore, MA, USA) was then applied for film development.

### Protein immunoprecipitation (IP)

CD8^+^GFP^+^ T cells sorted from *Cnr2*^*GFP*^ mice were washed with ice-cold PBS. IP lysis buffer supplemented with protease inhibitor and phosphatase inhibitor was directly added into cells and then lysed on ice for 15 min before cleared by 15,000 rpm at 4 °C. The proteins were then aliquoted equally into two samples. For IP of FLAG-tagged proteins, cell lysates were incubated with anti-FLAG M2 affinity gel (Sigma‐Aldrich, CA, USA) and mouse IgG magnetic beads (Sigma‐Aldrich, California, USA) on a rotator overnight at 4 °C. IP beads were then washed with IP lysis buffer three times and boiled in SDS loading buffer (2×) at 95 °C for 5 min for SDS-PAGE and immunoblot analysis.

### LC–MS/MS assay for protein identification

To identify the proteins interacting with CNR2, protein bands were displayed using silver-staining kit (Beyotime, Shanghai, China), Bands not detected in the IgG group were excised from the gels and performed LC–MS/MS analysis (Wininnovate Bio). Briefly, isolated protein samples were dried using a vacuum centrifuge, resuspended in 40 µl 50 mM ammonium bicarbonate, and prepared for LC–MS/MS. Tryptic peptide mixtures were separated using an Easy nLC UPLC system (Thermo Fisher, MA, USA) coupled with an in-house packed nanoViper C18 resin (3 µm, 100 Å) column (15 cm long 50 µm inner diameter). A 120-min gradient from 98% HPLC water/2% ACN/0.1% formic acid to 95% ACN/2% HPLC water/0.1% formic acid was used, and peptides were analyzed using a ThermoFisher Q Exactive MS system. Raw mass spectra were searched against a Uniprot Mouse Database and all MS/MS statistical analyses were performed utilizing PEAKS Studio 8.5 (version 8.5, Bioinformatics Solutions Inc. Waterloo, Canada) software.

### Statistical analyses

Statistical analyses were performed using the GraphPad Prism software (version 8; GraphPad Software Inc, San Diego, CA) and statistical significance was determined by *p* < 0.05. Comparisons between two groups were made using an unpaired Student’s T-test or one-way ANOVA. Two-way ANOVA was used for multiple comparisons. For comparing mouse survival curves, a Log-rank (Mantel–Cox) test was used. RNA-seq data statistical analysis was performed with R unless otherwise specified.

### Study approval

All mice were maintained under specific pathogen-free conditions and in accordance with the animal experimental guidelines of Sun Yat-sen University. All animal procedures were approved by the Institutional Animal Care and Use Committee of Sun Yat-sen University (L102012018002J). The Ethics Committee of Sun Yat-sun University Cancer Center approved the study of patient biospecimens (GZR2018-045).

## Supplementary information

Supplementary Materials

## Data Availability

The datasets used and/or analyzed during the current study are available from the corresponding authors on reasonable request.
