# Supplementary material for: Cannabis suppresses antitumor immunity by inhibiting JAK/STAT signaling in T cells through CNR2
Source: Signal Transduct Target Ther. 2022 Apr 6;7:99. doi: 10.1038/s41392-022-00918-y (PMC8983672; doi:10.1038/s41392-022-00918-y)
Supplement: Supplementary file 1 — Supplementary Materials [file 41392_2022_918_MOESM1_ESM.docx]

Supplementary Materials for

Cannabis Suppresses Antitumor Immunity by Inhibiting JAK/STAT Signaling in T Cells through CNR2

Xinxin Xiong^1,2^, Siyu Chen^3^, Jianfei Shen^4^, Hua You^5^, Han Yang^6^, Chao Yan^7^, Ziqian Fang^1^, Jianeng Zhang^1^, Xiuyu Cai^1^, Xingjun Dong^1^, Tiebang Kang^1^, Wende Li^3,*^, and Penghui Zhou^1,*^

Correspondence to: Penghui Zhou, [zhouph@sysucc.org.cn](mailto:zhouph@sysucc.org.cn); Wende Li [lwd@gdlami.com](mailto:lwd@gdlami.com)

**This PDF file includes:**

Figures. S1 to S6


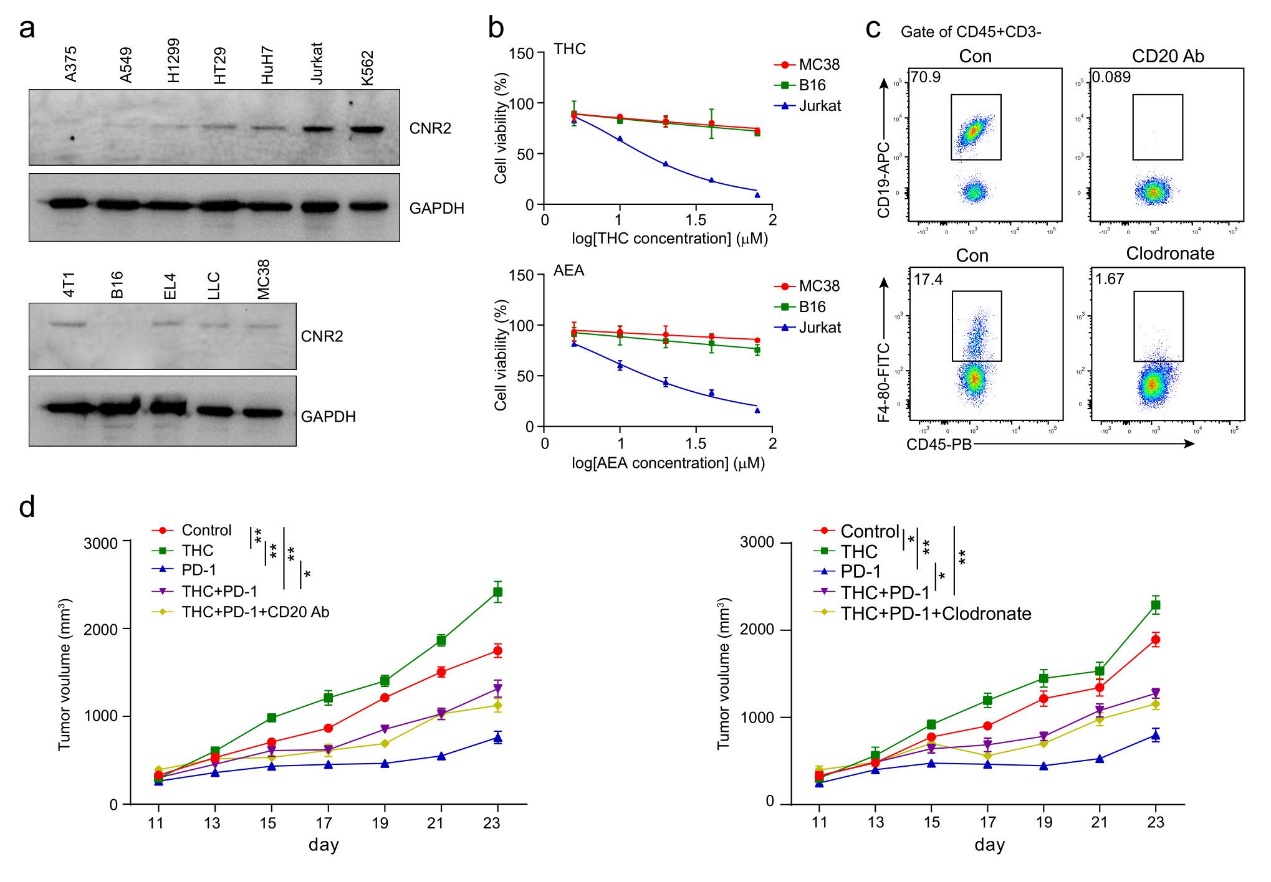


**Supplementary Fig. S1. THC-mediated suppression of antitumor immunity is not through tumor cells, B cells and macrophages.**

**a** CNR2 protein expression in tumor cell lines. Lysates (50 μg protein/lane) from tumor cells were analyzed by immunoblot using antibodies to either CNR2 or GAPDH. **b** Cell viability of B16, MC38 and Jurkat cells treated with various concentrations of THC and AEA, assessed by CCK8 assay. **c** *In vivo* administration of mAb SA271G2 and clodronate liposome depleted B cells and macrophages. **d** Mice bearing B16 tumors were treated with DMSO, THC, PD-1 Ab, THC+PD-1 Ab or THC+PD-1 Ab+ B cells depletion (left) or THC+PD-1 Ab+macrophage depletion (right) on day 10 after tumor inoculation. Tumor volumes were measured every other day (two-way ANOVA, mean ± SEM, *P<0.05, and **P<0.01).


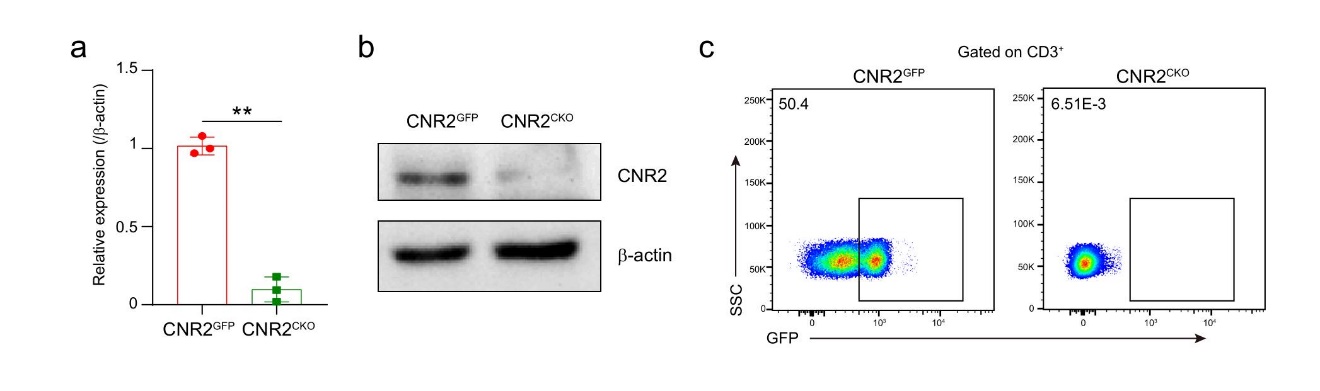


**Supplementary Fig. S2. Characterization of *Cnr2^CKO^* mice**

**a,b** qPCR analysis of *Cnr2* cDNA, and CNR2 immunoblot using CD8^+^ T cells from *Cnr2^GFP^* and *Cnr2^CKO^* mice (mean ± SD, **P < 0.01). **c** Flow cytometric analysis of the frequency of GFP^+^ populations in CD3^+^ T cells from *Cnr2^GFP^* or *Cnr2^CKO^* mice. Data are representative of three independent experiments.

**
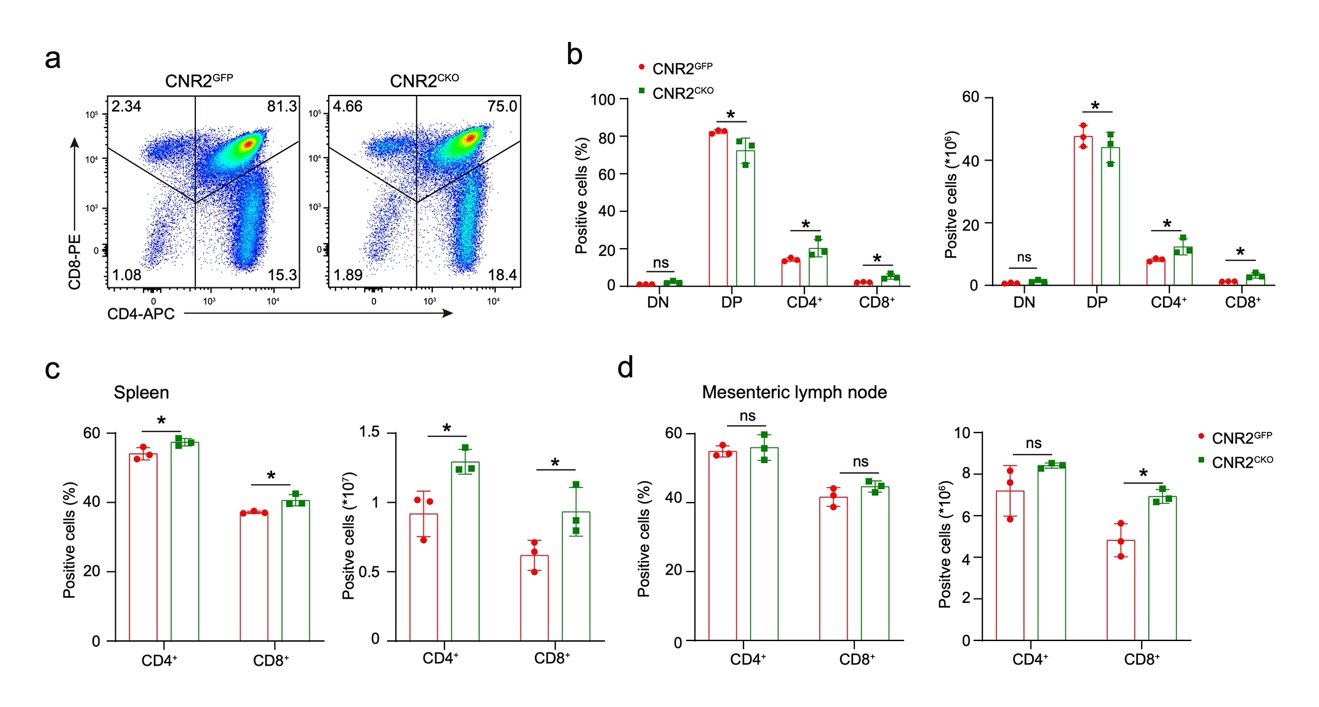
**

**Supplementary Fig. S3. Subsets of T cells in the thymus and periphery of *Cnr2^CKO^* mice**

**a** Gating strategy used to define single-positive CD4^+^ and CD8^+^, double-positive (DP), and DN thymocyte populations of *Cnr2^CKO^* and *Cnr2^GFP^* mice. **b** Quantification of thymocyte populations from **a**. **c,d** Percents and numbers of CD4^+^ and CD8^+^ T cells from spleen or mesenteric lymph node of *Cnr2^CKO^* and *Cnr2^GFP^* mice. Statistical significance was assessed by two-way ANOVA, mean ± SD, *P < 0.05 and **P < 0.01. ns, no significance, P> 0.05.


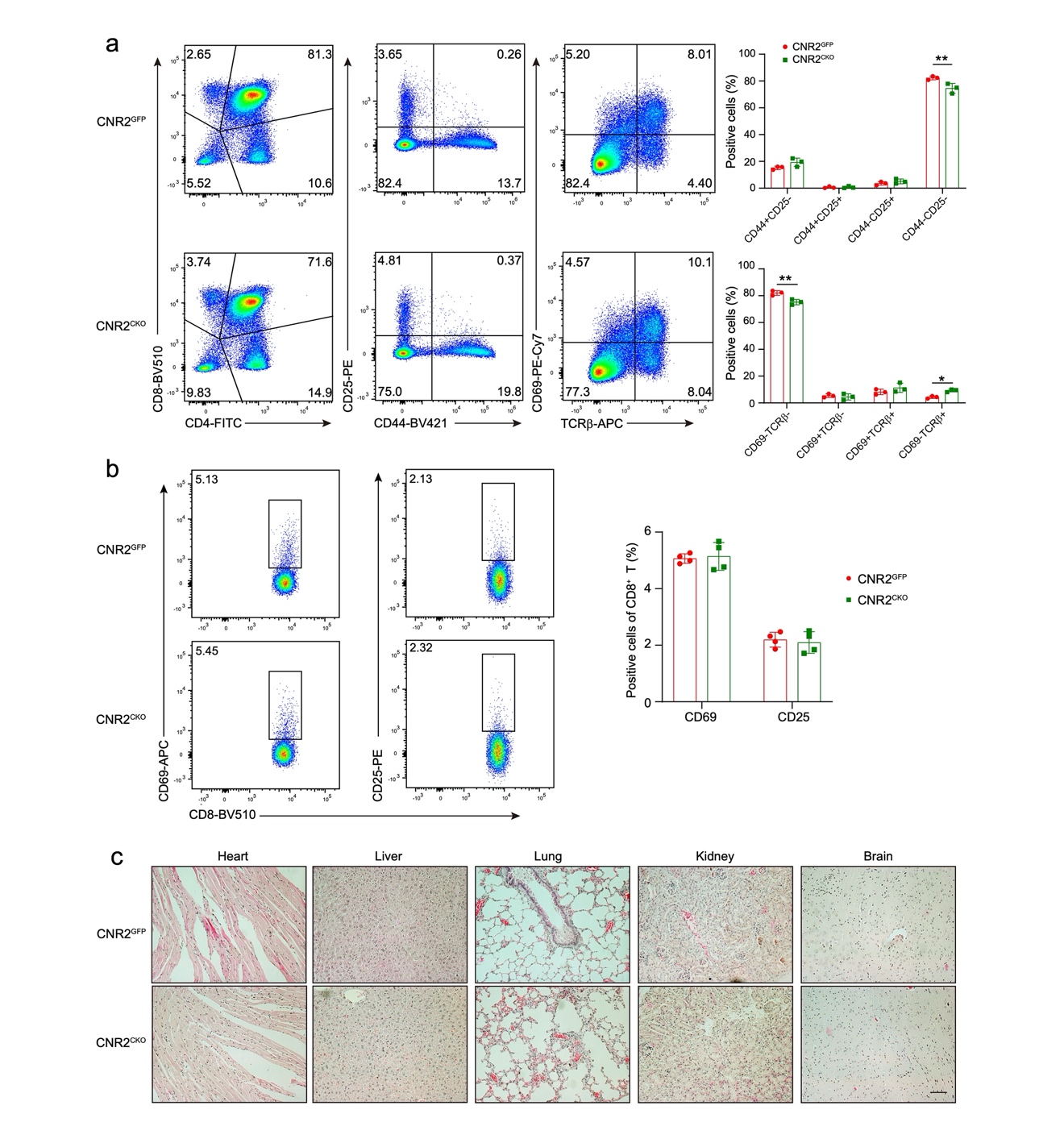


**Supplementary Fig. S4. Development of T cells in *Cnr2^CKO^* mice**

**a** Flow cytometry analysis of the surface expression of CD4, CD8, CD25, CD44, CD69 and TCRβ on *Cnr2^GFP^* and *Cnr2^CKO^* thymocytes; quantification of each subsets was shown at right. **b** Flow cytometry analysis of the surface expression of CD25 and CD69 on T cells from spleens of aged *Cnr2^GFP^* and *Cnr2^CKO^* (left); quantification of each subsets was shown at right. **c** H&E staining of the heart, liver, lung, kidney and brain from aged *Cnr2^GFP^* and *Cnr2^CKO^* mice. Statistical significance was assessed by two-way ANOVA, mean ± SD, *P < 0.05.


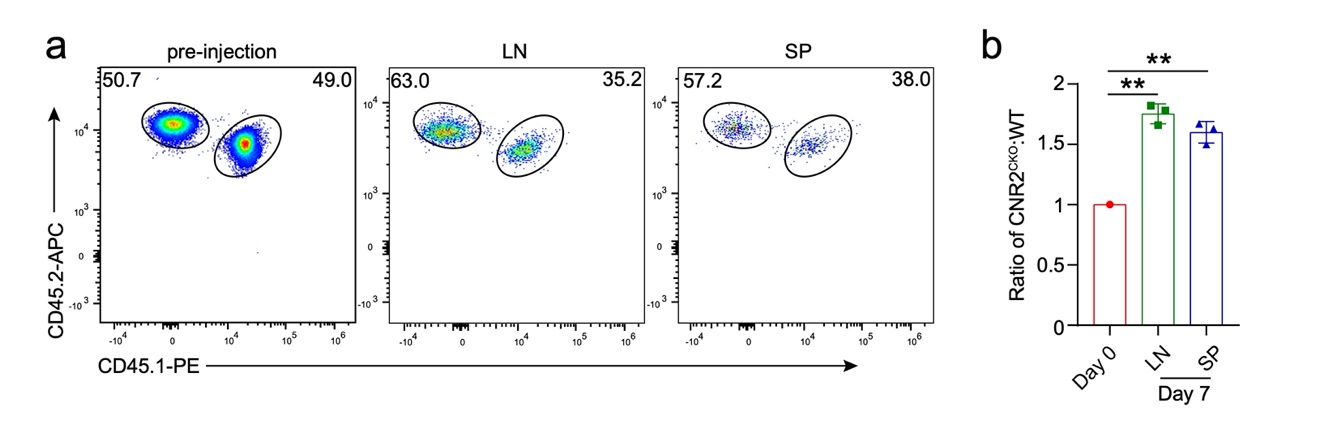


**Supplementary Fig. S5.** **Increased homeostasis of *Cnr2* deficient T cells**

**a** *Cnr2^GFP^* (CD45.1^+^CD45.2^+^) and *Cnr2^CKO^* (CD45.2^+^) CD8^+^ T cells were 1:1 mixed and intravenously injected into Rag2^-/-^ mice (CD45.1^+^). Flow cytometric analysis shows the frequencies of *Cnr2^CKO^* and *Cnr2^GFP^* CD8^+^ T cells from lymph node and spleen. **b** Representative ratios of *Cnr2^CKO^* to *Cnr2^GFP^* CD8^+^ T cells in the lymph node and spleen evaluated on day 7. Statistical significance was assessed by two-way ANOVA, mean ± SD, **P < 0.01.


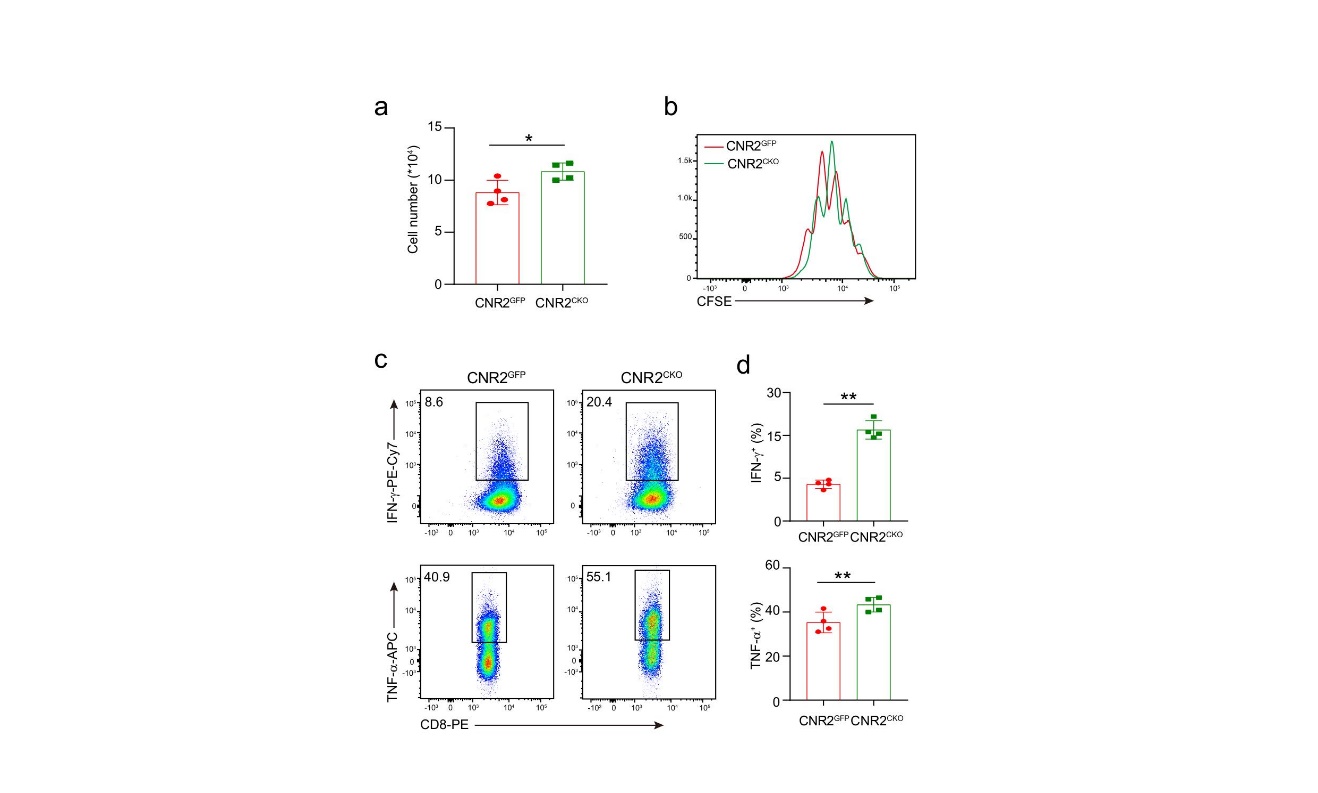


**Supplementary Fig. S6. *Cnr2* deficiency enhances T cell function and proliferation**

CD8^+^ T cells from *Cnr2^GFP^* or *Cnr2^CKO^* mice were treated with anti-CD3 plus anti-CD28 for 48 hr. **a** Number of *Cnr2^GFP^* or *Cnr2^CKO^* CD8^+^ T cells after stimulation (mean ± SD, *P < 0.05). **b** Proliferation of *Cnr2^GFP^* and *Cnr2^CKO^* CD8^+^ T cells measured by CFSE dilution assay (mean ± SD). **c,d** IFN-γ and TNF-α production in *Cnr2^GFP^* or *Cnr2^CKO^* CD8^+^ T cells measured by flow cytometry analysis (mean ± SD, **P < 0.01). Statistical significance was assessed by two-tailed unpaired Student’s t test (**a, d**). Data are representative of three independent experiments.
